# Proceedings of the Medical Society of the State of Pennsylvania

**Published:** 1853-06

**Authors:** 


### PROCEEDINGS OF THE MEDICAL SOCIETY OF THE STATE OF PENNSYLVANIA,

### *At its Annual Session, May,* 1853.

Pursuant to the Resolution passed at the last Session, the State Medical Society of Pennsylvania assembled in Philadelphia, on Wednesday 25th of May, 1853, at Sansom Street Hall, the place designated by the Committee of Arrangement of the Philadelphia County Delegation.

The President, Dr. Hiram Corson, of Montgomery, called the Society to order, and stated that the first business would be the appointment of a Committee to examine and verify the Credentials of the Delegates. Drs. Wilson Jewell, of Philadelphia, J. Bernard Luden, of Huntingdon, and Patrick Cassidy, of Lancaster, were appointed this committee, who, after some time, reported that they had examined the credentials submitted, and found them duly authenticated, with the exception of those from Delaware, which were not dated.

Dr. R. K. Smith, of Delaware, stated that correctly dated credentials were incorporated in the Report of the Delaware County Medical Society, and that the date had been inadvertently omitted in the certificate handed in.

Dr. Jewell mentioned that a communication had been presented to the Committee on Credentials from the Mifflin County Medical Society, appointing Drs. Ard and Rothrock delegates to the State Society, but the communication was not signed by the President or Secretary of the Mifflin County Society.

On motion of Dr. H. S. Patterson, of Philadelphia, the communication was received and the delegates were acknowledged.

The following are the delegates whose credentials were presented. [Those marked * were absent during the session.]

*Alleghany*—Drs. John Dickson,* Wm. M. Gray,* John Pollock,* T. W. Shaw,* T. J. Gallaher,* Chas. F. Williams,* George McCook,* Wm. M. Herron,* Daniel McNeal,* John Wilson.*

*Berks*—Drs. John P. Hiester, Wm. Herbst,* C. H. Wanner, J. Seiberling, *Martin Luther, F. M. Hiester.

*Bradford*—Dr. George F. Horton.

*Blair*—Drs. James Coffey,* J. M. Confer.

*Bucks*—Drs. Ralph Lee,* Isaac Newton Evans, Samuel*Thompson, Charles W. Smith,* Samuel Scott,* James S. Rich.*

*Cambria*—Drs. John Lowman,* James L. How.*

*Chester*—Drs. Wilmer Worthington, Isaac R. Walker, Samuel H. Harry, Septimus A. Ogier, J. P. Edge, Jacob Price, Charles W. Parrish,* Edward F. Rivinus,* W. D. Hartman,* J. H. Bradford.*

*Delaware*—Drs. R. K. Smith, C. J. Morton, J. Owen, Geo Martin, C. Ash.

*Erie*—Drs. W. Poyntell Johnson,* W. B. Williams,* Llandaff Strong,* H. A. Spencer.*

*Huntingdon*—Drs. J. B. Luden, J. M. Gemmill, J. A. Shade,* G. H. Winterode.*

*Lancaster*—Drs. Francis S. Burrowes, Emanuel S. Baer, J. L. Zeigler,* J. L. Atlee, George Hoover, Thomas Ellmaker, Benjamin Rohrer,* Henry Carpenter, Benjamin Musser,* J. Augustus Ehler,* Patrick Cassiday, Ely Parry, M. A. Withers, J. Gibbons.

*Lycoming*—Drs. Thomas Wood, James Rankin.

*Mifflin*—Drs. B. Ard,* A. Rothrock.

*Montgomery*—Drs. John Schrack, Benj. Johnson,* William Corson, Geo. W. Wimley, E. K. Beaver, F. P. Poley, H. S. Jacoby,* Henry Geiger, Hiram Corson.

*Northampton*—Dr. Samuel Sandt.

*Philadelphia*—Drs. Wm. Ashmead, John B. Biddle, Henry Bond, Thomas F. Betton, J. R. Bryan, Chas. F. Bibighaus, Benj. H. Coates, Joseph Carson, D. Francis Condie, Wm. Darrach, Gouv. Emerson, James V. Emlen, George Fox, Paul B. Goddard, David Gilbert, Edward Hallowell, S. L. Hollingsworth,* Isaac Hays, N. L. Hatfield, Samuel Jackson, Wilson Jewell, E. Janvier,* Alf. L. Kennedy, Wm. H. Klapp, W. L. Knight, John P. Lamb, R. J. Levis, William Mayburry, John K. Mitchell, George W. Norris, Arnold Naudain,* Henry S. Patterson, Lewis Rodman, Neville C. Reid, Moses B. Smith, A. E.

Stocker, Laurence Turnbull, Francis West, Joseph Warrington, Thos. S. Yardley, J. V. Patterson,* John M. Pugh.

*Schuylkill*—Drs. Enos Chichester, John G. Koehler, B. F. Shannon, James S. Carpenter, Charles M. Steinberger, George Halberstadt,* Jacob F. Treichler.

*Susquehanna*—Drs. B. Richardson,* E. L. Park.*

On motion of Dr. Condie, of Philadeiphia, the reading of the minutes of the last session was dispensed with.

On motion of Dr. Lamb, of Philadelphia, the minutes of the last session were referred to a Committee, with a view to bring before the Society any unfinished business from the last session. Drs. Mayburry, of Philadelphia, Worthington, of Chester, and Lamb were appointed this Committee.

The President now read the Annual Address, prepared by him in pursuance of a resolution of the last session. He commenced by a feeling allusion to the death of a prominent member of the Society, Dr. Isaac Parrish, who was well remembered as an active participator in the proceedings of the last and every previous meeting of the Society, and who had a few months since been removed by death. Dr. Corson proceeded to descant upon the objects which had been aimed at and accomplished in the formation of the Society. To effect a thorough organization of the profession of the State, was one great object which had brought them together. But, they had also higher and nobler objects than mere self-aggrandizement,—the improvement of medical science, by bringing together the stores of facts and observations which were collected from year to year. He alluded in complimentary terms to the advantages to be derived by the physicians from the country in their intercourse with the profession and medical institutions of Philadelphia, while they from the country brought in their accounts of the diseases and epidemics as they prevailed in their respective localities, with reference to the geological formations, meteorological conditions, habits of life, and other circumstances under which they occurred. They might perhaps be enabled to present some valuable data of the modifications of the disease which prevailed in our State in the mining, the manufacturing, and the agricultural districts.

Dr. Corson next turned to the effects of the various forms of quackery, prevailing in our midst, on the advancement of medical science. He thought that, perhaps, we might learn something indirectly from each of them.

Passing to the subject of medical education, Dr. Corson dwelt with considerable emphasis on the neglect of chemistry in the curriculum of medical studies, and on its high importance to medical science. It was comparatively slighted in our medical schools, and he coincided with an opinion expressed by Prof. Hare, at the meeting of the American Medical Association, in 1847, that no student should receive a diploma without the vote of the Professor of Chemistry.

Dr. Corson thought it would be much better to ask the Medical Colleges to insist upon a knowledge of chemistry as essential to graduation, than to require private practitioners to shut their office-doors upon those youths who were anxious to enter upon the study of medicine, but were ignorant of Latin and Greek. If we asked for too high a standard of preliminary education, we might exclude youths of genius and ennobling qualities, who had not had the advantages of superior early education—such self-made men as Franklin, Rittenhouse, Velpeau, and the elder Parrish.

On the subject of the County Medical Reports, Dr. Corson expressed a wish that attention might be turned to the use and abuse of some of the mineral medicines. He paid his respects rather pointedly to mercury, and hoped that future reports from the County Medical Societies would take up this subject, and give them information as to the diseases in which mercurials were essential, and those which were curable without them.

Dr. Corson concluded his address with some very strong remarks on the employment of alcoholic liquors by medical men. He was not only severe in his denunciation of their dietetic and social use, but he took the ground that "alcoholic stimulants are unnecessary in the treatment of any disease."

On the termination of Dr. Corson's address, Dr. H. Carpenter, of Lancaster, moved that the thanks of the Society be tendered to Dr. Corson for his eloquent address, just delivered, and that a copy be requested for publication.

Dr. Jewell could not vote for this resolution, if the sentiments contained in the Address were to go abroad as those of the Society. There was no more decided temperance man than he, but he was not prepared to sanction the opinion that we can dispense entirely with the use of alcoholic stimuli in the treatment of disease. He thought Dr. Corson's views ultra. He could not support the printing of the address as it had been delivered.

Dr. H. S. Patterson did not consider the Society in any way committed to the views of the Address.

And Dr. H. Carpenter, in moving to print the Address, had no idea of expressing any endorsement of the opinions advanced in it.

The resolution was adopted.

Dr. West, of Philadelphia, presented his report as Treasurer of the Society.

On motion of Dr. Condie, it was referred for examination to a Committee, consisting of Drs. Burrowes, of Lancaster, Wood, of Lycoming, and Bibighaus, of Philadelphia.

Dr. Condie presented the report of the Committee of Publication. 750 copies of the Transactions of last year had been printed, of which 500 had been sent to County Societies, and 136 disposed of, and sent to journals, &c.; making in all 636 distributed, and leaving 114 on hand. The publication had cost $305, other expenses $3.62; making an entire cost of $308.62. From County Societies $145 had been received; from the Treasurer $135.97; from Dr. Innes, for extra copies, $5; from Dr. La Roche, for the same, $1. A balance of $21.62½ remained due the Committee of Publication. The assessments from the counties of Huntingdon, Lebanon, Lycoming, Mifflin, Perry and Susquehanna, ($35)

had not been received. He was told, though, that the Huntingdon county assessment was here and would be handed in, and also that there was a letter in the Post Office containing that from Susquehanna.

On motion of Dr. Condie, the Commtttee on Publication was authorized to draw on the Treasurer for $21.62½.

On motion of Dr. Condie, it was resolved that the daily meetings of the Society shall be from 10 A. M. to 1 P. M., and from 4 to 6 P. M.

Dr. West extended an invitation to the Society to visit the Mint tomorrow morning at 9 A. M.

On motion of Dr. Atlee, of Lancaster, Dr. Burnell, of Massachusetts, was invited to a seat on the floor.

On motion of Dr. Jackson, of Philadelphia, Dr. Spencer, of Syracuse, was invited to a seat on the floor.

Dr. Beaver, of Montgomery, moved that Dr. Geiger be admitted as a substitute for five of the Montgomery county Delegation, who were absent.

Dr. Lamb asked if Dr. Geiger was a resident of Montgomery. And, it being stated that Dr. Geiger was a resident of Philadelphia, Dr. Fox, of Philadelphia, thought it not proper that he should represent Montgomery.

Dr. R. K. Smith, of Delaware, wished the question settled, if a county can be represented by a delegate not residing therein, also, if, when a member of a County Society moves out of a County, his association with the County Society is not dissolved. The Delaware county Medical Society desired information on this point.

Some discussion took place on this question, and finally, on motion of Dr. Condie, the subject was referred to a Committee, to report at the afternoon session. Drs. Condie, Koehler, of Schuylkill Haven, and Maybury were appointed this Committee.

On motion the Society adjourned.

*Afternoon Session.*

The President called the Society to order at 4 P. M.

The President presented the acknowledgment of the Society of the State of New York for the Transactions of the Pennsylvania State Society, and also the Transactions of the Medical Society of the State of New York.

The roll was now called. The minutes were read and approved.

Dr. Jewell reported that Dr. Samual Sand had been appointed a delegate from Northampton County Medical Society, in place of Dr. C. C. Field.

Dr. Jewell reported that Dr. Henry Geiger had been appointed a delegate as substitute for Dr. McEuen, from Montgomery county.

Dr. Jewell reported that Dr. Confer, from Blair county, had been appointed a delegate from that county. He had been in the city a week, and his credentials had not reached him.

On motion of Dr. Worthington, Dr. Confer was admitted a delegate from Blair.

Dr. Warrington asked, with reference to the assessment, if the expenses of the State Medical Society, independently of the accommodations here, were not to be borne by the County Medical Societies *pro rata* to the number of delegates.

Dr. Warrington moved a resolution to this effect.

Dr. Worthington, of Chester, seconded the motion.

Dr. Condie said, that unless delegates now came forward and paid an assessment, the publication of the Transactions would be delayed. The County Societies were backward in paying their contributions because many of them met so seldom. The assessment now to be levied would meet the more urgent pecuniary demands to be made on the Publication Committee, while the contributions of the County Medical Societies would complete the demand.

Dr. Warrington thought that the principle of throwing the burden of these expenses on the delegates was unjust, and he hoped the practice would now be established of making the County Societies bear this expense.

Dr. Patterson read the article of the Constitution providing that County Societies pay the expenses of publication according to their ratio of representation. The State Society to fix the rate of assessment.

Dr. Warrington moved that a Committee be appointed to fix the rate.

Dr. Condie thought it was impossible that a Committee could make in advance a rate of assessment which would be proper, as they could not tell what would be the cost of the Transactions. He thought it better that the delegates should pay an assessment now, and collect it afterwards from the County Societies. He moved the assessment be $2 on each of the delegates.

Dr. Atlee begged leave to say a word. He thought Dr. Condie's opinion, from his long experience in publication, entitled to great deference. But he must still deem it unjust that delegates who came here at their own expense, should be taxed for the cost of the Transactions in addition. They were besides to be taxed in their own Societies *pro rata.* Lancaster county sent a large delegation, who would be all taxed here, and then taxed *pro rata* in their County Societies. Thus a large delegation is most largely taxed. The more zeal shown in a county, the more the pecuniary burthen.

Dr. Warrington reiterated his views, which were that the County Societies be taxed according to the number of delegates to which they are entitled, whether sent or not.

Dr. Fox thought that this was all provided for by the Constitution. All monies should be received and distributed by the Treasurer. He had resigned the office of Treasurer because this had not been done. The Chairman of the Committee of Publication had received and paid out money without previously handing it to the Treasurer. The proper mode was for County Societies to hand over to the Treasurer the money they collect, and then for the Committee of Publication to draw on the Treasurer.

Dr. Worthington thought it would be proper for the Treasurer or the Chairman of the Committee on Publication to make an assessment on County Societies, when the cost of publication was known, and he was sure the amount called for at once would be transmitted to the Treasurer. Treasurer and Publication Committee would confer together and fix ratio and assessment. County Societies were bound to return lists of their members. He moved the Treasurer make assessment on County Societies, *pro rata* for representation. Let them know what it is and have it transmitted.

Dr. Burrowes, of Lancaster, thought the Publication Committee could not go on without funds in hand. It would be better to get some money now from delegates.

Dr. Worthington's motion was now carried.

Dr. Condie, from the Committee to whom was referred the question whether members of County Societies, moving out of the County, forfeit their membership, reported: According to a strict construction of the Constitution this would be the result; but it would defeat the object of bringing within the organization of the profession every member in good standing. A physician may move to a place where there is no Society, or some time may elapse before he can connect himself with the Society where he has moved, or he may be nearer the place of meeting of an adjoining county than his own. The Committee recommend a certain latitude to county Societies as regards these cases. They recommend that physicians moving into another county, in good standing, be received into the Society of the county where they move, on certificate from county left.

Dr. Atlee said the rule in Lancaster county is to strike from the roll all who move permanently from the county. He thought it a good rule. To change it would be to reverse one of the great principles on which the State Medical Society was founded, viz. the organization of the profession. The County Societies should be entitled only to representation on their resident members. If a man goes to a county where there is no Society, let him go to work and organize a Society.

Dr. Atlee moved to recommit the report.

Dr. Worthington moved that the report be laid on the table, and that it be

*Resolved*, That the Society adheres to a strict construction of the Article of the Constitution which confines membership of County Societies to residents within the County. Carried.

Dr. Condie now moved that the State Society recommend to the County Societies to incorporate within their by-laws an article admitting members from other counties on a certificate from the county which they leave.

Dr. Worthington objected to this, as defeating the free action of the County Societies, and fettering them. Each Society could regulate its own membership.

Dr. Atlee moved to amend Dr. Condie's motion by making a man who removes merely *eligible* without a year's residence, but still let him be ballotted for.

Dr. Jackson opposed Dr. Condie's resolution.

Dr. H. Carpenter moved that Dr. Condie's resolution be laid on the table. Carried—24 to 20.

Dr. John P. Heister, of Reading, from Berks County Medical Society, laid before the Society the following report and resolutions on adulterated drugs, from Berks County Medical Society:

"After carefully considering the able and valuable reports made by the different Committees on adulterated drugs, appointed by the Medical Association, your Committee would respectfully suggest that there appears to be still a considerable want of system in the modes proposed, of abating or abolishing the serious evil of the manufacture and sale of spurious drugs, so loudly and justly complained of. The nearest approach to a feasible, and in the opinion of your Committee, effectual plan, is that suggested by Dr. R. M. Huston, and reported in the Transactions of the Association for 1851.

On an attentive review of the whole subject, being deeply impressed with its great importance, and aided and guided by the valuable suggestions contained in the reports of the Committees of the Association above referred to, your Committee would most respectfully submit the following resolutions, to be taken up by your delegates to the next session of the State Medical Society for its consideration:

1. *Resolved*, That the County or local Societies of this State be urgently requested to appoint annually committees to collect samples of drugs from dealers and manufacturers, in their respective districts, to be presented to the State Medical Society at their annual sessions.

2. *Resolved*, That the Medical Society of the State of Pennsylvania appoint annually a Committee of three to take charge of the said samples, and it shall be their duty to employ a competent assayer of drugs to examine the same, and give an opinion on their purity and quality, and this opinion shall be presented to the Society for publication in their Transactions.

3. *Resolved*, That the amount of expense incurred by the Committee in carrying into effect the above resolutions, shall be assessed and collected in like manner as is assessed and collected the amount for defraying the expenses for publishing the Transactions.

Dr. Heister advocated these resolutions at some length.

Dr. Parry, of Lancaster, thought the plan expensive and impracticable.

Dr. Turnbull, of Philadelphia, expressed the same views.

On motion of Dr. Biddle, of Philadelphia, the resolutions were referred to a Committee to inquire into the expediency and practicability of carrying out the suggestions embodied in the Berks County resolutions, and to report to the next session of the Society. Drs. Biddle, Parry and Carson, of Philadelphia, were appointed this Committee.

The President presented an invitation from the Academy of Natural Sciences to visit their Hall at any hour designated by the Society.

The invitation was accepted for Friday afternoon at 3.

Dr. H. Carpenter moved that a committee of one delegate from each county be appointed to nominate officers for the ensuing year. Carried.

Dr. Condie moved that the order of the day for to-morrow, immediately after reading the minutes, be Reports from County Societies.

The nominating Committee appointed, consisted of—

| | | |
|---|---|---|
| Dr. H. Carpenter, | . . . | Lancaster, |
| " C. H. Warner, | . . . | Berks, |
| " G. F. Horton, | . . . | Bradford, |
| " Isaac Newton Evans, | . . | Bucks, |
| " Isaac R. Walker, | . . . | Chester, |
| " R. H. Smith, | . . . | Delaware, |
| " J. D. Luden, | . . . | Huntingdon, |
| " Thomas Wood, | . . . | Lycoming, |
| " A. Rothrock, | . . . | Mifflin, |
| " J. W. Wimley, | . . . | Montgomery, |
| " Samuel Sands, | . . . | Northampton. |
| " D. Gilbert, | . . . . . | Philadelphia, |
| " John G. Koehler, | . . . | Schuylkill, |
| ———— | | Susquehanna. |

Dr. Burrowes, from the Committee to audit the Treasurer's account, reported that they examined and found it correct. Report accepted.

The hour of adjournment now arrived.

*Thursday—Second day—26th of May.*

The Society was called to order by the President at 10 o'clock A. M.

The roll was called, and the minutes read and approved.

Reports from County Societies being in order, the Report of Sanitary Committee of the Medical Society of the City of Reading of the County of Berks, for 1853, was presented and read by one of the Secretaries. A prevalence of typhoid fever and a typhoid tendency in diseases generally, an epidemic of parotiditis in a district of the county, were among the most noticeable points in this report.

Dr. Wm. Gries, Chairman of the Committee, expressed an unfavorable opinion of the use of alkaline and saline preparations, where an hemorrhagic tendency prevails in typhoid fever. His experience was also decidedly against the general use of alcoholic stimulants in this epidemic. He found the use of aromatic sulphuric acid very advantageous, both in restraining the hemorrhagic tendency and alvine discharges, and in supporting the patients. The sulphate of quinia, which Dr. Gries had in previous epidemics employed with advantage, he derived no advantage from in this, except in the declining stages of the fever, when it occasionally presented something of an intermittent character.

The Report from the Chester County Medical Society was next read. The report was necessarily brief, it was stated by Dr. C. W. Parrish, chairman, as only five communications had been received, and none intimated the prevalence of any general epidemic. The physicians from whom communications had been received, were Drs. Worthington, Hartman and Price, of West Chester, Edge, of Downingtown, and Pennypacker, of Phœnixville. Dr. Edge, of the Chester Valley, reported the occurrence of numerous cases of typhoid fever in his district.

He thought the disease had been clearly communicated from patient to patient. Its appearance in Uwchlan, north of the Valley, was traceable to the scattering of the pupils of a boarding school, where the fever had prevailed. Dr. Edge himself felt the effects of the disease in increased languor, &c., for two weeks.

Dr. Hartman reported several cases of dysentery in the mica slate ridge, the same district in which it occurred in 1851. He had tried the nitrate of silver, and his experience was quite unfavorable as regards its effects.

Drs. Worthington, Pennypacker, Price and Hartman, all referred to a disease, as prevailing from last of January to last of spring months, which was probably influenza, although catarrhal symptoms were not prominent. Dr. Pennypacker found sulphate of quinia very useful in managing it. Dr. Worthington had treated it by evacuants very successfully.

A few cases of erysipelas, cholera morbus, cholera infantum, rubeola, &c., were recorded. Pertussis had occurred epidemically in certain districts.

Dr. Pennypacker had met with an unusual number of cases of acute rheumatism, some of them of great violence.

This Report and the preceding were referred to the Committee on Publication.

An invitation to visit the Academy of Fine Arts was now presented, and accepted for one o'clock to-day.

An invitation from Rev. Dr. Ducachet, of St. Stephen's Church, to visit the Statuary in that Church was received, and one o'clock on Friday was fixed for accepting the invitation.

The Secretary now read the Report from the Schuylkill county Medical Society. Dr. James S. Carpenter, the Chairman, stated that he had received no communications from the physicians of the county. No epidemics had prevailed in the county. Since the occurrence of the great flood of 1850 in the Schuylkill and its tributaries, a marked diminution in the occurrences of miasmatic fevers had been noticed. A few cases of typhoid fever had been observed, traceable, according to Dr. Carpenter's views, to defective drainage. He had used sulphate of quinia and nux vomica with good effect. A tendency to cerebro-spinal meningitis had been noticed. In the treatment of dysentery he had used tincture of chloride of iron with much success. In scarlatina, he had used inunction in conjunction with chlorate of potassa internally, with very striking advantage.

Dr. Carpenter mentioned two cases of strangulated hernia, in which, after every known means of reduction had been attempted, the bowels was readily and promptly reduced by inhalation with *chloroform.*

The influence of *altitude* upon disease Dr. Carpenter briefly alluded to. He stated, that within certain limits, elevation of situation and the consequent rarefaction of the atmosphere, he believed to be conducive to a healthy condition of the lungs. Dr. Carpenter intimated an intention on the part of the Schuylkill County Medical Society to devote more attention to this subject in a future report.

Report referred to Committee of Publication.

Dr. R. K. Smith now read the sanitary report of the Delaware County

Medical Society. Scarlatina, rubeola, variola, dysentery, miasmatic fevers, cutaneous and rheumatic fevers had been the prevalent diseases. It was observed that the districts affected with miasmatic fever were exempted from dysentery; and also that the districts affected this year with dysentery had been exempt the year before. Dysentery had taken a very malignant and fatal form in the onset of the epidemic. Geological formation appeared to exercise very little influence over it. As regards treatment, calomel and opium, or some other form of mercury, castor oil, laxatives, (more or less frequently) with laudanum or oil of turpentine, acetate of lead and ipecacuanha, with opium, were found of great value; Hope's mixture, anodyne and astringent enemata, were also resorted to by many. 28 cases and 2 deaths, and 30 cases and no deaths, are the numbers recorded in two reports.

Dr. Smith stated that the physicians of the northern portion of the County had not responded to the circular issued, and were consequently unrepresented in the report.

Dr. H. Carpenter read the Report of the Lancaster County Medical Society. This report contained an elaborate and interesting geographical, geological and statistical description of the County. The report was accompanied by a chart. As regards diseases of past year, but little is to be offered, it being a period of unusual health; and only one communication was received in reply to the circulars issued. Dysentery had been most prevalent in slaty or primary districts. The surrounding country was entirely exempt. As regards non-prevalence of dysentery in limestone regions, mentioned in last year's reports of several Counties, the Lancaster County Report cannot concur. The dysenteric epidemic appeared to be of a miasmatic origin, and required a corresponding treatment. Total number of practitioners in the County 155; graduates 102; non-graduates 28; total regular practitioners 130.

This Report was also referred to Committee on Publication.

The Report from Blair County was read by the Secretary. Only two reports had been received in this County, besides those furnished by members of the Committee. The Committee commented upon the negligence thus evinced.

Rubeola, scarlatina, variola, varicella and varioloid, typhoid fever, and dysentery, were the diseases noticed, with a remarkable absence of ordinary, acute and inflammatory fevers, and miasmatic diseases. The past year had been one of general health. The Report furnished very interesting and full details of the various diseases which had been noticed. Drs. Confer, Ross, and others, contributed voluminous reports to the Blair Co. Society. Dr. Landis of ——— county, was attacked by typhoid fever, from exposure, fatigue &c., incident to his duties among his patients with that disease. The writer denies the contagious character of the disease—its production owing to deliberating agents generally—believes it to be epidemic—has never seen a case where ventilation and other preventive measures were fairly used. Has met with more obstinate cases of neuralgia during past year than he can recollect of previously—has on the whole been a year above the average state of health. Topographical and Geological reports will be made in another year. Medical Society in existence seven years—held four meetings in 1852—Drs. Coffee and Confer, a committee to prepare reports for 1852—officers—members—named—referred.

Report of Mifflin co. read by Dr. Rothrock; County has been unusually healthy—sporadic cases tending to typhoid type; typhoid pneumonia rather common—some sporadic cases of dysentery—(reading here suspended—some of the manuscript being mislaid—to be resumed when found.)

Huntingdon co. Report read by Dr. Luden. Has also enjoyed unusually good health. Rubeola, varicella, the only epidemic during past year still prevails at close of year, mostly in the borough of Huntingdon. Writer believes typhoid fever communicable by contagion—mortality from said fever during this year about 9 per cent.; prevailing mostly upon the slaty and wet districts. Dr. Miller reports cases of dysentery, mostly upon the slaty formation; yet some very severe ones occurring on the limestone formation. The cases of intermittent, and remittent, less than other years. Neuralgia of every variety more frequent than of late years. Small pox prevailed to some degree; mortality about 7 per cent. Anthrax, furunculi, &c., very common. Inflammatory disease of respiratory organs rather numerous during the winter months; some putting on the typhoid type. Referred to committee of publication.

Dr. Atlee of Lancaster, Chairman of committee of epidemics of Pennsylvania, New Jersey, Maryland, of the National Medical Association, read a communication on the subject, calling the attention of the members to the subject, and urging the members to make observations and communicate them to him. Referred to the committee of publication, to be published with the minutes. On motion adjourned.

*Afternoon Session.*

The President called the Society to order at 4 o'clock, P. M. Roll called. Minutes read and approved.

A communication, read before Huntingdon County Medical Society, on Small-Pox, Vaccination, and Varioloid, by Dr. Gemmill, was presented as an appendix to Huntingdon county report. It was read, and referred to Committee on Publication.

Dr. Condie stated that, owing to the ill health of the Chairman of the Committee of the Philadelphia County Medical Society, Dr. H. S. Patterson, the report from Philadelphia county was not prepared. He moved, that if ready before the publication of the transactions, it be included in them.

The report of the Susquehanna County Medical Society was prepared by Dr. Latham, chairman, which was presented and read. It contained a topographical and geological description of the county, and a brief sketch of the diseases which had prevailed during the past year. The general health of the county had been remarkably good, and nothing worthy of especial notice had occurred, except an epidemic of a mild form of scarlatina. Referred to Committee of Publication.

Dr. Jewell extended an invitation to the Society from the Philadelphia delegation, to an entertainment to be given in the hall this evening.

On motion adjourned to visit Girard College.

*Third day—Friday—27th of May.*

The President called the Society to order at 11 o'clock.

Dr. Cassiday, from the Committee on Credentials, reported those of Cambria county delegates correct.

The minutes of the last meeting were read and approved.

Dr. Maybury, from the Committee on Unfinished Business of the last session, reported.

On motion of Dr. Patterson, the report was accepted. The Committee was continued, and the committees named in the report were called on for their respective reports.

Drs. Carpenter and Halberstadt, of Schuylkill, extended an invitation to the Society to hold its next meeting at Pottsville.

On motion of Dr. Condie, it was resolved that the next meeting of the Society be held at Pottsville.

The report of the Cambria County Medical Society was read and referred.

The report of the Montgomery County Medical Society was now read. It presented a very full and interesting account of the diseases observed in this county, with full details of the treatment employed. Dr. H. Geiger was the Chairman of this Committee.

The report of the Bucks County Medical Society was now read. No regular report was made, but an account was submitted of epidemics which prevailed in the county some years ago, which they asked to be referred to the Committee of Publication, with discretionary power to print the whole or part.

Dr. Walker, of Chester, offered the following resolution, which was adopted:

*Resolved,* That it be the duty of the Recording Secretaries to transmit to the Chairman of the Committee of Arrangements of the "National Medical Association," a correct list of the delegates from this Society, so that it may be received at least one week previous to the meeting of said Association. Also, that the Secretaries shall supply each delegate with a certificate of his appointment.

Dr. West offored the following resolution, which was adopted:

*Resolved,* That the Committee of Publication be instructed to prefix to the forthcoming volume of the transactions of this Society, a note, or statement, that the Medical Society of the State of Pennsylvania does not endorse or sanction the facts or opinions contained in any address or report which may be made to them, or published by their authority.

Dr. R. K. Smith offered the following resolution:

*Resolved,* That each Medical Society be requested to furnish with their reports a synopsis of their contents, and that the reports be read by their titles, with the synopsis of their contents, and referred to the Committee of Publication.

Dr. Warrington, of Philadelphia, was opposed to the indiscriminate publication of the reports coming from various quarters. He thought that the right of selection, condensation, curtailment, or rejection, should be vested in the Committee of Publication, with reference to reports.

Dr. Condie said this Society was not organized as a censorship over men's minds or their productions. He wished every man to present his facts and views in his own way. The object of the Society had been to rouse up the dormant energies of the profession of the State, and he thought it would be very unwise to reject the materials furnished in the reports because men might take exception to portions of them. How many differences of opinion existed in every branch of medicine—physiology for instance? Who should undertake to say what was correct and what was not? Freedom of mind was essential to the advancement of science. The moment you undertake to trim and curtail the reports you will cease to receive reports. He was for receiving all the reports and printing them as they were received, while it would be stated in the Transactions that the Society in no way endorsed or sanctioned the opinions and facts presented in them. He felt that the Medical Society of Pennsylvania might be proud of their transactions as already published.

Dr. R. K. Smith thought the resolution of Dr. West, just passed, met every objection that might be felt to opinions advanced in reports. He was opposed to any curtailment or alteration in the reports.

Dr. Emerson, of Philadelphia, wished that the reports should always be accompanied by abstracts, not exceeding eight foolscap pages, which should be read in place of the reports, unless they were of unusual interest. He moved, as an amendment to Dr. Smith's resolution, that each report be accompanied by a synopsis not exceeding eight post pages.

Dr. Geiger, of Montgomery, concurred in the views expressed by Dr. Warrington. He thought there was a great deal of lumber in the reports—matter to be found in all the text books, or copied from Rogers' Geological Manual—that they should not be at the expense of publishing.

Dr. Jackson said that there had not been a medical book published since the days of Hippocrates, that did not contain the lumber alluded to by the gentleman who spoke last. He did not feel the force of his objections.

Dr. Hays thought we might legislate too much. He thought it unwise to put upon the county Societies the additional labor of furnishing abstracts, and, besides, he considered it disrespectful to ask the county Societies to make reports, and then to decline to read them in full.

Dr. Corson took the floor, and expressed himself strongly opposed to any interference with the reading of the reports in full. He believed that they were doing incalculable good in rousing the energies of the heretofore isolated country practitioners, in teaching them to think and judge for themselves, and to throw off the trammels of the teachers whose opinions and dogmata, however valuable, had hitherto been too blindly followed. He was for receiving and reading the reports in full.

Dr. J. K. Mitchell, of Philadelphia, said, that the great objection to allowing any committee to cut to pieces the reports, was that what some men would call lumber, others might think jewels. He had often derived great satisfaction from the old ideas and opinions which were revived in these reports, and which some gentlemen deemed so valueless. He was against any cutting up of these Reports. He was for the fullest array of facts in these reports. He thought it an omission in some of them to have omitted the age at which death took place in fatal cases. It had an important bearing in the mortality of many diseases, typhoid fever for instance.

Dr M. B. Smith, of Philadelphia, was opposed to cutting down the reports.

On motion of Dr. Walker, of Chester, both resolutions were indefinitely postponed. Yeas, 28, nays, 7.

Dr. Burrowes, of Lancaster, offered the following resolutions, which were unanimously adopted:

*Whereas,* Since our last meeting it has pleased Providence to remove from us our friend and professional brother, Dr. SAMUEL DUFFIELD. As a father, friend and physician he had few superiors. To see was to know him: he looked, thought and talked like a gentleman. His removal has left a vacancy not easily filled; in him "cum tristibus severe, cum remissis jucunde," was not the result of artificial accommodation.

*Resolved,* That the Pennsylvania Medical Society receive with unfeigned regret the intelligence of the death of their firm friend and professional brother, Dr. DUFFIELD.

*Resolved,* That a copy of these resolutions be transmitted by the Secretary to the family of our lamented brother, expressive of our heartfelt sympathy.

Dr. West offered the following resolutions, which he prefaced by feeling and eloquent remarks:

*Whereas,* Since the last meeting of this Society, it has pleased Almighty God, in his wise Providence, to remove by death from our midst, Dr. ISAAC PARRISH, a most useful and highly valued member of the Society;

*And whereas,* it is due to his memory, as well as useful to those whom he has left behind, to hold up for esteem and imitation his purity of purpose and great integrity of character, both private and professional, as well as the constant devotion and zeal exhibited in his efforts to advance the common cause of medical science, medical education and medical ethics; therefore,

*Resolved,* That the members of the Medical Society of the State of Pennsylvania, have received, with feelings of unaffected sorrow, the intelligence of the death of Dr. PARRISH, which occurred shortly after the last annual session of the Society, and that they desire thus to record their high estimation of his talents and personal worth, as well as their sense of the heavy loss sustained in his death, by surviving friends both in and out of the profession, as well as by the community at large.

*Resolved,* That a copy of these resolutions be transmitted by the Secretary to the family of the late Dr. Parrish, with the assurance of the sincere sympathy of the Society in their bereavement.

Dr. H. Carpenter offered the following resolutions:

*Resolved*, That this Society deeply regrets the decease of its late fellow member and first President, Dr. SAMUEL HUMES, of Lancaster city, for whose moral worth, high professional character, and active zeal in promoting the objects and interests of this organization, the members of the State Medical Society will ever fondly cherish his memory.

*Resolved*, That the Secretaries are hereby instructed to transmit a copy of the above to the friends of the deceased.

Dr. West stated that the memoir of Dr. Parrish by Dr. Jackson could be obtained by application to the Secretary.

Dr. Jewell called for the reading of the Report of the Bucks County Medical Society.

Dr. Condie was unwilling to compel the reading of this Report, as the authors did not desire it.

Dr. Thompson, of Bucks, said that the communication in question was not a Report of the Bucks County Society, but merely an account of an epidemic, apparently diphtheritis and typhoid fever, which prevailed in Bucks County some six years since. He had no objection, however, to the reading.

And it was accordingly read by the Secretary.

After the reading, Dr. Jewell said he was opposed to publishing mere individual Reports, unless they receive the sanction of the County Medical Society.

Dr. Emerson concurred.

Dr. Condie said this was substantially a County Report. The author of the Report, Dr. Thompson, said it was not a County Report.

Dr. Emerson moved to reconsider the vote by which this communication was referred to the Committee of Publication, and ordered to be published; but the motion was lost.

Dr. H. Carpenter brought in the Report of the Nominating Committee, and the officers therein nominated for the ensuing year were unanimously elected. The following are the officers:

*President.*—Dr. John P. Heister, of Bucks.

*Vice-Presidents.*—Drs. Francis F. Burrowes, of Lancaster; John H. Gemmill, of Huntingdon; Robert K. Smith, of Delaware; Jacob M. Confer, of Blair.

*Recording Secretaries.*—Drs. Henry S. Patterson, of Philadelphia; Isaac R. Walker, of Chester.

*Corresponding Secretary.*—Dr. Isaac Hays, of Philadelphia.

*Treasurer.*—Dr. Francis West, of Philadelphia.

*Censors, 1st and 2d Districts.*—Drs. Wilson Jewell, of Philadelphia; Wm. Corson, of Montgomery; George Halberstadt, of Schuylkill; Samuel A. Benton, of Delaware; S. Thompson, of Bucks.

*Censors, 3d and 4th Districts.*—Drs. Thomas Wood, of Lycoming; Mordecai Massey, of Huntingdon; A. Rothrock, of Mifflin; Wm. W. McIlvain, of York; J. H. Case, of Perry.

*Censors, 5th and 6th Districts.*—Drs. C. F. Perkins, of Erie; J. McK. Morgan, of Alleghany; J. P. Gazzam, ditto; Jas. Dickson, do.; W. Anderson, ditto.

*Delegates to the American Medical Association for* 1854.—Drs. Hiram Corson, of Montgomery; Wm. Maybury, of Philadelphia; John L. Atlee, of Lancaster; Trail Green, of Northampton; J. S. Carpenter, of Schuylkill; Edward Wallace, of Berks; Isaac R. Walker, of Chester; John L. Ross, of Blair; —— Irwin, of Alleghany.

Dr. N. B. Smith proposed a vote of thanks to Mr. Schæffer, who furnished the geological map appended to the Schuylkill County Report.

Unfinished business from the last session being called for—

The Committee to urge the Legislature to pass an act for general vaccination being called in, reported progress and asked to be continued.

Dr. Atlee urged the vast importance of this subject. He thought the object might be accomplished by insisting that no child should be admitted into common schools, unless with a saticfactory certificate of vaccination. In Prussia, no child could be admitted to a public school, or confirmed by the minister, or bound out an apprentice, unless with such a certificate. Some such plan as this was the only means of accomplishing the object.

Drs. Emerson and Condie stated that the petitions already presented to the Legislature intimated the means by which the object could be carried out.

Dr. Atlee was added to the Committee.

The Committee to whom was referred a plan investigating the connection between geological formations and disease, reported that the only practicable plan for accomplishing this object, would be to analyse the County Reports presented from year to year.

Report was accepted, and Committee discharged.

Dr. Heister moved that County Societies be requested to furnish topographical descriptions of their respective counties, accompanied by charts, and to describe epidemics of disease, with particular reference to the geological formations. Carried.

Adjourned.

*Friday May 27th.—Afternoon Session.*

The Association met at 4 o'clock; the President, Dr. Corson, in the Chair.

The roll being called, the minutes of the morning session were read.

Dr. Condie requested the minutes to be amended, relative to a motion stated to be made by him, but which should be really attributed to Dr. Burrowes.

The minutes were adopted.

Dr. J. L. Atlee offered the following resolution, which was adopted:

*Resolved,* That the Committee on Publication be requested to furnish annually, to the Epidemiological Society of London, a copy of all published Transactions of this Society.

Dr. Robert K. Smith offered the following resolution, which was adopted:

*Resolved,* That the thanks of this Society be tendered for the polite invitation received to visit the Academy of Natural Sciences, Academy of Fine Arts, U. S. Mint, Laurel Hill Cemetery, Statuary of St. Stephen's Church and Girard College; and to Pres. Allen, of the last institution, for his attention and hospitality to the members of this Society, on the occasion of their visit.

Dr. Condie presented the following resolution, which being put by the Secretary, was passed:

*Resolved*, That the thanks of this Society be presented to Dr. Hiram Corson, its late President, for the impartial and courteous manner in which he has presided over its deliberations during the present session.

Dr. Turnbull offered the following resolution, which was adopted:

*Resolved*, That the thanks of this Society be presented to the Secretaries for the faithful manner in which they have discharged their laborious duties.

Dr. Parry offered the following, which were adopted:

*Resolved*, That, for the purpose of acquiring further information in relation to the Kine Pock, and increasing facilities for procuring and preserving supplies of fresh and genuine vaccine virus, this Society recommend to each of the County Societies in the State to appoint a Committee to collect any new and useful facts in relation to vaccination, and to report the same to a central Committee, to be appointed by this Society, at least two weeks before the annual meeting.

*Resolved*, That said central Committee shall consist of three members, and it shall be its duty to draw up a synopsis of the reports received, and present the same to this Society, at its annual meeting.

The Chair appointed Drs. E. Parry, H. Carpenter and P. Cassiday a Committee under the above resolution.

Dr. S. Jackson, of Northumberland, in order to expedite and cheapen the printing of the Transactions, offered the following, which was passed.

*Resolved*, That it is recommended to each County Society to send by its delegates, to the State Society, one dollar for every five of the members of said County Society, to be paid to the Treasurer.* Each County Society to pay the balance of its quota on the delivery of the Transactions.

Dr. Heister offered the following resolutions, which were carried:

*Resolved*, That this Society earnestly requests all County Societies that have not yet done so, to furnish, at an early period, topographical histories of their respective counties, accompanied by charts.

*Resolved*, That the annual reports of the different County Societies be recommended to have strict reference to the different geological formations, in order to ascertain, by a comparison of facts, how far the prevalence of epidemics and other diseases may be influenced by them.

Dr. Turnbull suggested the propriety of asking the Legislature to pay for the printing of the Transactions of the Society.

After some discussion by Drs. Atlee and Condie, Dr. Coates requested Dr. Spencer, of New York to give his views upon this matter.

Dr. Spencer said, that the manner in which they conducted these

* No Society, however small, to send less than five dollars.

matters in New York was very plain and simple. Having fully made up their minds as to the propriety of what they were about to ask for, they then endeavored to influence the Legislature, by appealing individually to its older members, and explaining to them the practical benefits to the community that were sure to result from such a publication.

Dr. H. S. Patterson called the attention of the Society to certain resolutions which had been laid on the table at the last yearly meeting, and which, he stated, now came up to be acted upon.

Dr. Maybury moved that the resolutions offered by Dr. Emerson, at that meeting, be adopted. Agreed to.

Dr. Condie moved that the resolutions of the Montgomery County Society, laid on the table at the last yearly meeting, be indefinitely postponed. They were so postponed.

On motion, Drs. Condie, Norris and Yardley, were added to the Publication Committee.

Dr. —— moved that the President-elect be requested to deliver an address at the opening of the next annual meeting. He was so requested.

Dr. J. L. Atlee moved that a Committee be appointed to conduct the President-elect to the Chair.

Drs. Atlee and Maybury being appointed a Committee, conducted the President-elect to the Chair; when he was introduced to the Society by Dr. Corson.

Dr. Corson, the President, returned his thanks, upon retiring from the Chair, for the courtesy and kindness with which he had been honored by the Society.

Dr. Heister, of Berks County, the President-elect, said that when he looked around and saw so many, who were his seniors, he felt, indeed, highly honored by his present position. He took great interest in the organization of the profession throughout the whole United States, as well as in this State. He was among the founders of this Society, and his interest in its proceedings was yearly increasing, as, indeed, he believed, was that of all the members present. He was glad to see a still greater increase in the numbers of the Society, there being eighty-five present, an increase of ten over last year. In conclusion, he stated, that, with the kind assistance of the members, he would endeavor faithfully to fulfil the duties of his office.

Dr. Atlee offered the following resolution, which was adopted:

*Resolved*, That the thanks of this Society be presented to the Committee of Arrangements for their attention in furnishing such excellent accommodation for the session of the Society, and for the zeal manifested to provide for our comfort and pleasure.

Dr. Geiger, of Schuylkill County, moved that the next meeting of the Society be held in Pottsville, on the last Wednesday in May, 1854, at 11 o'clock in the morning. Adopted.

Dr. Corson moved that the thanks of the Society be tendered to Dr. Spencer, for the donation of a volume of the Transactions of the State Society of New York; and that a Committee of three, of which the President of the Society should be Chairman, be appointed, to take into consideration Dr. Spencer's atomic theory of vegetable and animal life.

Dr. Heister, the President, declined serving as Chairman.

Dr. Geiger moved that the President be excused from serving.

Dr. Corson moved that Drs. Turnbull, Kennedy and Carson be appointed.

Dr. Carson declined.

Dr. Kennedy also declined.

After some discussion, Dr. Coates moved for a reconsideration of Dr. Corson's motion. Being put, it was so reconsidered.

Dr. Coates then moved that the first clause of Dr. Corson's resolution be put by itself, which being done, was adopted.

Dr. Corson then withdrew the second clause of his resolution.

There being no further business before the Society, Dr. Atlee moved an adjournment *sine die.*

